# Clinical and Epidemiological Aspects of Parkinson's Disease in the South of Western Siberia

**DOI:** 10.3389/fneur.2020.538782

**Published:** 2020-11-03

**Authors:** Alexey Sergeevich Rozhdestvensky, Roman Andreevich Delov, Elena Andreevna Marks, Ivan Aleksandrovich Gaponenko, Elena Vladimirovna Khanokh

**Affiliations:** ^1^Department of Neurology Continuing Professional Education, Omsk State Medical University, Omsk, Russia; ^2^Omsk State City Hospital No. 7, Omsk, Russia

**Keywords:** Parkinson's disease, Siberia (Russia), hyposmia, cognitive impairment, depression

## Abstract

**Background:** The article is devoted to one of the most common neurodegenerative diseases in the world—Parkinson's disease (PD), the prevalence of which in Russia reaches 140–150 people per 100,000 people. The clinical and anamnestic profile of a patient with PD is presented, the prevalence of motor and non-motor symptoms is reflected, and a comparative characteristic of the neurological deficit in the Siberian population of patients with other cohorts of patients with Parkinson's disease in different countries and ethnic groups is presented.

**Methods:** We studied 140 patients with Parkinson's disease. A comprehensive assessment of neurological status was performed using the “Unified Parkinson's Disease Rating Scale (UPDRS).” In addition, we used the Beck Depression and MoCA scale test. Assessment of the presence and severity of olfactory dysfunction was performed using the Sniffin Stick odor identification test. The stage of PD was evaluated according to the classification of M. M. Hoehn and M. D. Yahr.

**Results:** The cohort of the study was dominated by overweight patients with a higher level of education, with concomitant arterial hypertension, coronary heart disease, and dyslipidemia. The severity of motor and most non-motor symptoms directly correlates with the duration of PD and the stage of the disease. The predominant form of the disease was a mixed form, which was also noted in research cohorts in Canada and the UK. The Siberian cohort tends to be more prevalent in hyposmia, daytime sleepiness, orthostatic hypotension, and depressive and REM disorders.

**Conclusion:** Our data show the importance of a comprehensive assessment of both motor and non-motor neurological deficits as well as the analysis of comorbid disorders and risk factors for the occurrence and progression of Parkinson's disease. They also show the prevalence of certain motor and non-motor symptoms in the Siberian cohort of patients with Parkinson's disease.

## Background

Parkinson's disease is a chronic progressive brain disease mainly associated with the degeneration of dopaminergic neurons of the substantia nigra with intraneuronal accumulation of the α-synuclein protein and the formation of intracellular inclusions (Lewy bodies). These are manifested by a combination of hypokinesia with rigidity, resting tremor, and postural instability, as well as a wide range of non-motor manifestations (mental, autonomic, sensory, etc.) (1–3).

Parkinson's disease is classified as a predominantly motor disorder, although clinical non-motor polymorphism in recent years has been the subject of extensive research (4–6). This is explained by a significant influence of non-motor symptoms on the quality of life, the progression of disability sometimes exceeding the negative effects of motor neurological deficit (7–9).

To date, there are no specific instrumental or laboratory markers of this disease that could be reliably used in everyday clinical practice. Despite the rapid scientific and technological advances in medicine, the diagnosis of PD in routine practice remains clinical and relies on the identification of cardinal motor signs of parkinsonism (hypokinesia, rigidity, rest tremor, and postural disorders) and the absence of atypical symptoms. In relation to this, a rigorous study of the clinical features and characteristics of the course of the disease is extremely important and is the key to success in a complex diagnostic process.

The study of the features of the course of various progressive diseases, including Parkinson's disease, is relevant in connection with the climatogeographic features of the Omsk Region, located in the south of the West Siberian Plain. The geographical location features open this territory for the interference of air masses: northern Arctic, warm southern Central Asian, dry western Central Asian, and cold eastern. The different nature of the air flow leads to sharp changes in temperature, making the whole weather of the region unstable. The climate of Omsk is continental temperate. According to https://world-weather.ru, obtained during long-term observations of air temperature, it is possible to present all the features of the climate in the Omsk region:

The lowest average temperature is −16.9°C (in January).

The highest average temperature is +18.9°C (in July).

Absolute minimum is −50°C.

Absolute maximum is +40°C.

From these data, it can be seen how large the temperature fluctuations during the year: 40° between average values and 90° between the minimum and maximum. This is one of the features of the continental climate. Omsk is characterized by the predominance of clear sunny days even in the autumn-winter period—from 223 to 300 during the year. The cloudiest month is December; in May, the number of such days is minimal. These climatogeographic features may probably influence the course of chronic progressive diseases, such as Parkinson's disease.

In accordance with the urgency of the problem, a retrospective clinical and epidemiological assessment of the clinical course of Parkinson's disease in the south of western Siberia was performed at Omsk State Medical University.

In accordance with the urgency of the problem presented at the Omsk State Medical University, a retrospective clinical and epidemiological assessment of the clinical course of Parkinson's disease in the south of western Siberia was carried out. The aim of our study is to assess the impact of climatic, geographical, and ethnic factors on the course of Parkinson's disease in the Siberian cohort of patients.

## Methods

### Patients

All patients (Russians residing in the Siberian part of Russia) were diagnosed with PD at the Omsk State Medical University. All patients with PD were selected and studied according to the international Unified Parkinson's Disease Rating Scale (UPDRS) and Hoehn and Yahr scores (10, 11). The diagnosis of PD was based on the UK PD Brain Bank Criteria (12). In addition, the Beck Depression Scale and Montreal Cognitive Assessment (MoCA) test was used (13). The severity of olfactory dysfunction was performed using the Sniffin Stick odor identification test.

In this work, we evaluated the clinical and epidemiological features of the course of the disease in 140 patients with Parkinson's disease. A retrospective analysis of medical documentation and an assessment of clinical parameters at the onset of the disease and at the time of the examination were carried out.

These were the criteria for inclusion of patients in the study:

- A reliable diagnosis of Parkinson's disease in accordance with the criteria of the European Federation of Neurological Societies (EFNS) in conjunction with the Movement Disorders Society 2013.
- Signed informed consent to participate in the study.
- The absence of other neurodegenerative diseases in the patient.

Criteria for exclusion of patients from the study:

- Secondary parkinsonism and parkinsonism—plus identified at the initial visit.
- The presence of severe concomitant somatic pathology in the stage of decompensation.
- The patient's refusal to participate in the study.
- Patient involvement in other clinical studies.
- The patient has other diseases that have a genetic component in the pathogenesis, due to the high risk of distorting the information received.

### Statistical Analysis

Descriptive statistics for qualitative accounting features are presented in the form of absolute values, percentages, their standard errors (m), and standard deviations (σ). Data for variational series with non-parametric distribution are described as medians and quartiles (Me [25th; 75th percentile]). For comparison of non-parametric data, the Mann–Whitney *U* criteria were used. The critical level of significance of the tests is determined at *p* ≤ 0.05. Statistical processing of the results was carried out using Statistica 10 licensed software packages (StatSoft, USA).

The study was approved by the Ethics Committee of the Omsk State Medical University.

## Results

According to inclusion/exclusion criteria, 140 patients with Parkinson's disease were included in the study, including 55 men and 85 women (the majority of patients had PD with the overage duration of 3–10 years with the symptoms effectively managed by combination of adequate treatment options (dopamine agonists, levodopa, amantadine). The ratio of men and women in the group was 1:1.5, with a predominance of females. The age of patients ranged from 37 to 82 years (median [25th; 75th percentile], 67 years [61; 73]): women, 85 (66.3 ± 9.5; age, 68 years [61; 72]), and men, 55 years (66.5 ± 9.7; age, 68 years [61; 74]). The average age of all patients at the time of the examination was 66.4 ± 9.5 years. Treated patients with PD received different medications (dopamine receptor agonists: pramipexole in a dosage of 1.5 mg/day or piribedil in a dosage of 150 mg/day, L-dopa in a dosage of 150–200 mg/day, and amantadine in a dosage of 300 mg/day), either as monotherapy or in various combinations.

The studied group of patients consisted of 14 (10%) Mongoloids and 126 (90%) patients of the European race. By the level of education, the group was divided into the following categories: 13 (9%) people had secondary education, 25 (18%) patients received secondary special education, and 102 (73%) patients graduated from higher educational institutions.

The average weight in the main group was 76.0 ± 14.3 kg (73.5 [65; 86]). The average height in the study group was 165.4 ± 8.0 cm (164.0 [160; 170]). Thus, the average body mass index is 27.8 ± 4.7 (27.0 [24.7; 30.7]), which indicates the predominance of patients with overweight.

The study conducted an analysis of risk factors for the development of PD and protective factors. The data are presented in Table 1.

**Table 1:** Risk/protective factors for patients with Parkinson's disease.

**Risk/protective factors**	**Patients with PD**	**Patients with PD (%)**
	**(abs)**	
Industrial contact with pesticides	8	5.7
Household contact with pesticides	44	31.4
Smoking	31	22.1
Quit smoking after the onset	6	4.3
Never smoke	103	73.6
Drink at least 1 cup of coffee per day	78	55.7
Do not drink coffee	62	44.3
Mild traumatic brain injury	14	10

The analysis of anamnestic information about the presence of concomitant diseases showed that patients with PD are quite comorbid and usually have several nosological forms in the structure of the diagnosis. The data are presented in Table 2.

**Table 2:** Comorbidity of patients with Parkinson's disease.

**Diseases**	**Patients with PD (abs)**	**Patients with PD (%)**
Ischemic stroke	4	2.9
Malignant neoplasms	3	2.1
Coronary heart disease	44	31.4
Arterial hypertension	55	39.3
Dyslipidemia	28	20
Diabetes	12	8.6
Hyperthyroidism	3	2.1

The presence of depressive symptoms was reported by 4 (2.9%) patients with PD, but depression of varying severity during the assessment of the Beck depression scale conducted in the framework of this study was detected in 46 (71%) of the 65 patients examined. The average level of depression in the group was 15.6 ± 9.1 points, which corresponds to mild depression.

## Motor Symptoms in the Siberian Cohort

In the study group of patients, the stage was determined according to the classification of Hen-Yar (1967): in 31 patients, one stage of the disease was established, in 63 patients, two stages, in 45 patients, three stages, and in one patient, four stages of the disease.

The average disease duration in the observed group of patients was 6.9 ± 4.9 years. The debut of PD with motor symptoms was noted in 129 (92.1%) patients, the debut with non-motor symptoms was detected in 11 (7.9%) patients with PD. Of the motor symptoms of the debut, the obligate symptom was hypokinesia, which was anamnestically established in all 140 (100%) patients. Other motor symptoms of PD debut in frequency were arranged in decreasing order as follows: resting tremor−76 (54.3%) patients, muscle rigidity−65 (46.4%) patients, and postural instability in PD debut was not noted.

In the analysis of the current clinical picture in patients with PD, hypokinesia was detected in 140 (100%) patients, resting tremor was diagnosed in 95 (67.9%) patients, rigidity in 97 (69.3%), and postural instability in 43 (30.7%) patients with PD.

Thus, the increase in the frequency of occurrence of the main motor symptoms of Parkinson's disease in the Siberian cohort over a 7-years period was 14% for resting tremor, 23% for muscle rigidity, and 31% for postural instability.

In our cohort of patients, 98 (70%) patients with a mixed form of the disease, 22 (16%) patients with a rigid-trembling form, and 20 (14%) patients with an akinetic-rigid form of Parkinson's disease were observed. We compared our data on motor deficiency with other cohorts of patients that were collected and systematized this year (14). The data are presented in Table 3.

**Table 3:** Motor subtype in *de novo* PD cases by country.

**Study**	**Year**	**Country**	**Total study participants**	**Mean Age (year)**	**Sex M/F**	**TD (No.)**	**TD (%)**	**Rigid akinetic or PIGD (No.)**	**Rigid akinetic or PIGD (%)**	**Indeterminate or mixed (No.)**	**Indeterminate or mixed (%)**	**Method of subtyping**
Reinoso	2014	Singapore	576	63.8	328/248	19	3.3	383	66.5	174	30.2	Lewis method and Rossi modifications
Rajput	2017	Canada	156	65.0	98/58	10	6.4	45	28.8	101	64.7	Novel method
Ramani	2016	UK	42	67.0	29/13	7	16.7	17	40.5	18	42.9	Novel method
Poletti	2011	Italy	42	65.0	28/14	10	23.8	24	57.1	8	19.0	Lewis method
Alves	2006	Norway	171	71.3	112/87	43	25.1	92	53.8	36	21.1	Jankovic
Auyeung	2012	Hong Kong	171	62.2	93/78	46	26.9	62	36.3	63	36.8	Novel method
Yuan	2013	China	51	61.9	24/24	20	39.2	19	37.3	12	23.5	Jankovic method with Korchovinov modifications
Mocciia	2016	Italy	63	60.6	38/25	27	42.9	18	28.6	18	28.6	Jankovic
Konno	2018	USA	1,003	64.0	637/366	439	43.8	386	38.5	178	17.7	Most prominent symptom at diagnosis
Seong-Min Choi	2018	South Korea	192	66.2	94/98	87	45.3	82	4.1	23	12.0	Jankovic
Muller	2011	Norway	207	67.9	122/85	95	45.9	88	42.5	24	11.6	Jankovic
Hiorth	2013	Norway	207	67.9	122/85	95	45.9	89	43.0	23	11.1	Novel UPDRS ratio
Nicoletti	2016	Italy	485	65.6	292/193	311	64.1	104	21.4	70	14.4	Most prominent symptom at diagnosis
Rozhdestvensky	2020	Russia	140	66,4	55/85	22	16	20	14	98	70	Most prominent symptom at diagnosis

Lewis method with Rossi modifications, ratio of the mean tremor scores (TD) (items 20 and 21) and the mean akinetic-rigid score (AR) (items 18, 19, 22, and 27–31): if the ratio TD/AR is >2.0, it was defined as TD and if AR/TD is more than 2.0, it was defined as AR. Mixed type was any indeterminate result. Jankovic: ratio of mean TD scores divided by mean of postural instability and gait items (falling, freezing, subjective gait difficultly, gait, and postural instability): if ratio is >1.5 TD, PD; if ratio is <1.0 PIGD, PD. Other subtyping methods are as described in the reference materials.

*TD, tremor dominant; PIGD, postural instability and gait disorder*.

Table 3 presents numerous studies of motor deficiency in Parkinson's disease in various ethnic groups (14–26).

## Non-motor Symptoms in the Siberian Cohort

The most frequent non-motor symptoms of PD debut in the study group were constipation in 47 (33.6%) patients, cognitive impairment in 24 (17.1%) of 140 patients, behavioral disturbances in the REM phase of sleep in 24 (17.1%) of patients, insomnia diagnosed in various variations in 38 (27.1%) patients, daytime sleepiness in 43 (30.7%) patients, subjective olfactory sensation dysfunctions at the time of the motor debut of PD in 19 (13.6%) patients, unscheduled weight loss in 8 (5.7%) patients, anhydrosis of the skin in 7 (5%) patients, sweating already in the debut of PD in 21 (15%) patients, seborrhea (indicated as an additional non-motor symptom of the debut of PD) in 4 (2.9%) patients, orthostatic hypotension in 35 (25%) patients, dysphagia in 5 (3.6%) patients, and a dysfunction of the pelvic organs by the type of night urination and fecal incontinence in 17 (12.1%) patients.

In the analysis of current non-motor symptoms, cognitive impairment was detected in 61 (43.6%) patients, psychotic disorders in the form of hallucinations in 7 (5%) patients, behavioral disturbances in the REM phase of sleep in 65 (46.4%) patients, olfactory dysfunction in 28 (20%), daytime sleepiness in 84 (60%), insomnia in various variations in 59 (42.1%) patients, unplanned weight loss in 12 (8.6%) patients, orthostatic hypotension in 80 (57.1%), dry skin in 6 (4.3%), hyperhidrosis in 29 (20.7%), dysphagia in 21 (15%), constipation in 85 (60.7%), and night urination in 41 (29.3%) patients.

**Figure 1:**
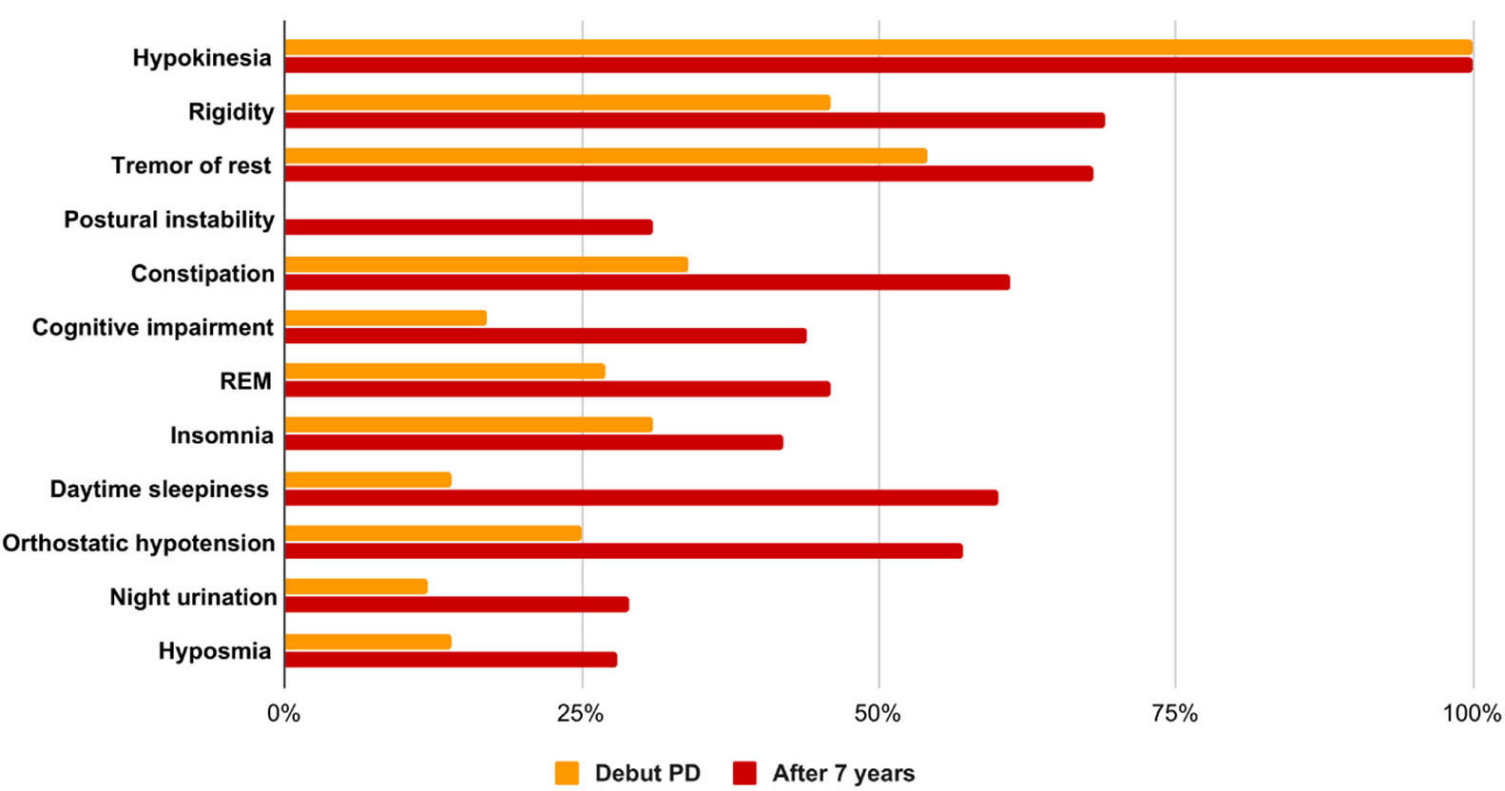
Features of the clinical picture in the debut of PD and at the time of the examination, taking into account the average duration of the disease.

Thus, the increase in the prevalence of non-motor symptoms of Parkinson's disease in the Siberian cohort for a 7-years period was constipation in 27%, cognitive impairment in 27%, REM disorders in 29%, hyposmia in 6%, orthostatic hypotension in 32%, insomnia in 15%, daytime sleepiness in 29%, and nocturia in 17%. BP progression over a 7-years period is shown in Figure 1.

**Figure 2:**
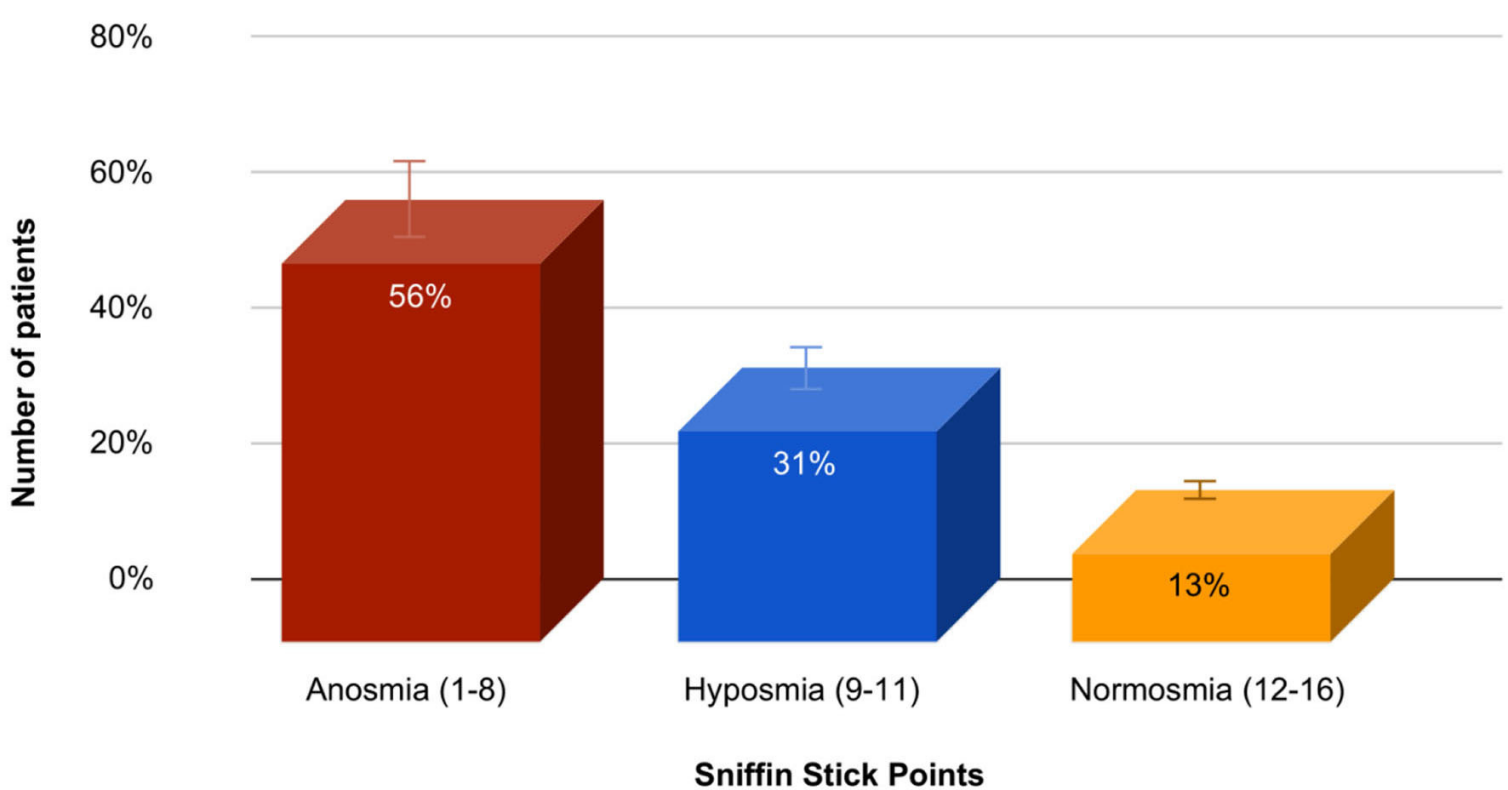
The prevalence and severity of olfactory dysfunction in the study group of patients with PD.

Olfactory test (Sniffin Stick) or Sniffin Sticks odor identification test (Bürghard firm, Hamburg, Germany) was performed in 83 patients, and the average score was 7.6 ± 3.3, which corresponds to anosmia. Identification test: the ability to detect odors from the proposed four names. The patient is given a pencil to inhale the smell and are offered four options, one of which he must choose. The result is the sum of the positive answers. A result of <8 points corresponds to anosmia, 9–11 points to hyposmia, and 12–16 points to normosmia. In this group of patients, olfactory dysfunction reaching the degree of anosmia was diagnosed in 47 (56.2%) patients, hyposmia in 26 (31.3%) patients, and normosmia in 11 (12.5%) patients. The data are presented in Figure 2.

A statistical analysis of a group of patients with Parkinson's disease at the onset of the disease and at the time of the study, taking into account the average duration of the disease course of 6.9 ± 4.9 years using the non-parametric Mann-Whitney *U*-test, showed significant differences in the severity of the main motor and non-motor symptoms. At the time of the study, patients had more pronounced resting tremor (*U* = 1,021.5; *p* < 0.0000001), general bradykinesia (*U* = 1,854; *p* < 0.001), and rigidity (*U* = 1,630.0; *p* < 0.0007). When comparing the severity of non-motor symptoms in the debut of BP and after 7 years, more pronounced constipation (*U* = 1,387.5; *p* < 0.000001), cognitive impairment (*U* = 1,683.5; *p* < 0.0002), behavioral disorders were noted in the REM phase of sleep (*U* = 1,775.5; *p* < 0.0004), insomnia (*U* = 1,082.5; *p* < 0.0000001), daytime sleepiness (*U* = 1,534.0; *p* < 0.000008), orthostatic hypotension (*U* = 1,826.0; *p* < 0.000008), and hyposmia (*U* = 836.0; *p* < 0.00004). There is also a tendency to increase the severity of night urination (*U* = 1,814.5; *p* < 0.02).

We compared the results of evaluating some non-motor symptoms in our cohort of patients with Parkinson's disease with symptoms in other cohorts of patients around the world; data for which were systematized this year (27). The data are presented in Table 4.

**Table 4:** Some non-motor symptoms in patients with PD by country.

**Study**	**Year**	**Country**	**Total study participants**	**Mean years since diagnosis**	**Anosmia (%)**	**Dysphagia (%)**	**Constipation (%)**	**Nocturia (%)**	**Weight change (%)**	**Memory problems (%)**	**Hallucinations (%)**	**Depression (%)**	**OH (%)**	**EDS (%)**	**Insomnia (%)**	**RBD (%)**	**Hyperhidrosis (%)**
Duncan	2014	UK	158	0.5	44	20	42	25	23	54	22	37	32	25	18	35	10
Romenets	2012	USA	70	3.8	21	16	30	68	21	42	12	38	38	14	41	38	19
Hui-juan Li	2015	China	82	5.1	45	33	67	87	29	95	15	67	38	73	78	52	65
Cosentino	2013	Peru	300	5.8	36	22	56	77	53	61	20	81	48	33	48	36	43
Khedr	2013	Egypt	112	6.2	10	24	52	60	33	30	13	47	54	39	46	15	21
Cheon	2008	S Korea	74	6.4	28	31	66	68	35	61	18	65	64	26	56	35	60
Rodríguez-Violante	2011	Mexico	232	6.6	34	33	58	62	28	47	19	67	46	28	47	33	39
Martinez-martin	2007	International	545	7	29	28	53	62	18	45	23	50	28	31	46	36	30
Tanveer	2018	Pakistan	97	7	26	28	60	77	38	59	30	52	53	41	53	36	37
Bostantjopoulou	2013	Greece	166	7.1	26	14	46	52	7	31	2	42	28	9	26	27	21
Chaudhuri	2010	UK, Germany, Spain	242	8	43	27	48	65	23	51	17	49	39	35	47	39	31
Rozhdestvensky	2020	Russia	140	6.9	56	15	61	41	9	44	5	75	57	60	42	46	21

Any data that was not available is replaced with a dash.

*OH, orthostatic hypotension; EDS, excessive daytime sleepiness; RBD, REM sleep behavior disorder*.

Table 4 shows the prevalence of major non-motor symptoms identified during studies in different corners of the globe (8, 28–37).

The average score during neuropsychological testing of the cognitive sphere using the Montreal scale for assessing cognitive functions was 23.4 ± 3.9, which corresponds to moderate cognitive impairment. A score of 20 and lower was scored by 13 (15.1%) patients, and in the range of 21–25 points, 43 (51.8%) patients were observed; the normal values of this test [i.e., 26–30 were noted in 28 (33.1%) patients with PD].

## Conclusion

Of course, factors such as ethnicity (38), geographical location of the region of residence (39), diet (40), overall life expectancy (41), genetic characteristics of the population (42, 43), and presence of concomitant diseases (44) affect the development and nature of the course of both neurodegenerative and other chronic progressive neurological diseases. A comparative assessment of the clinical parameters of Parkinson's disease in populations is to some extent difficult due to the variability of approaches for obtaining clinical data and their interpretation. In addition, the UK PD Brain Bank Criteria used in most studies provide a diagnostic accuracy of only 82.7% for the diagnosis of Parkinson's disease (45). This indicator, of course, affects the statistical reliability of clinical and epidemiological studies of Parkinson's disease and can lead to some distortion, especially in the early stages, when the clinical picture is usually incomplete, the response to dopaminergic drugs is uncertain, or signs of atypical parkinsonism have not yet appeared. Despite significant advances in understanding the pathogenetic aspects of this disease, we still lack clear imaging or biochemical markers to accurately diagnose Parkinson's disease.

In our work, we estimated the prevalence of motor and non-motor neurological deficits, as well as some risk factors and concomitant diseases in the Siberian cohort of patients with Parkinson's disease. The predominant debut of BP is the motor debut. The progression of the disease is characterized by an increase in the frequency of both motor and non-motor symptoms. When comparing the prevalence of motor symptoms with other cohorts of patients (Table 3), we found the greatest similarity with Canadian (24) and, to a lesser extent, UK (15) cohorts of patients, in which mixed forms of Parkinson's disease also prevailed. This similarity may be due to the close climatic and geographical features of western Siberia and Canada with a sharply continental climate, and the predominance of patients of the European race in our cohort. The analysis also noted the predominance of tremor in the structure of the clinical picture of Parkinson's disease in the Italian (14, 23), Norwegian (18, 26), and South Korean (16) cohorts. Against this background, a study of Reinoso et al., conducted in 2014, which shows the predominance of an akinetic-rigid form or postural instability with gait disorders of up to 66.5% in a cohort of patients with Parkinson's disease (21), looks quite interesting. The ratio of disease forms in the US (22) and China (19) cohorts was approximately equal.

In this article, we compared the prevalence of non-motor symptoms of Parkinson's disease in Siberian and other cohorts around the world. Based on these data, the Siberian cohort demonstrates a greater prevalence of olfactory disturbances, daytime sleepiness, orthostatic hypotension, depressive disorders, and behavioral disorders in the REM phase of sleep compared with cohorts similar in terms of the duration of Parkinson's disease (29–33, 35, 36). However, the prevalence of dysphagia, nocturia, weight changes, and hallucinations turned out to be lower than in the cohorts of patients similar in terms of sample size and duration of the disease. It is possible to consider these parameters only tentatively, since they have differences in the methodology for assessing non-motor symptoms, cohorts have ethnic differences, which probably affect the final result.

In the study group of patients, mild depressive symptoms, mild cognitive impairment, and severe olfactory impairment (anosmia) were noted.

The severity of motor and most non-motor symptoms directly correlates with the duration of PD and the stage of the disease, which is confirmed by most clinical trials of this disease (46–48).

The comorbidity of patients with Parkinson's disease is an urgent public health problem. A study by Mollenhauer et al. reflected the negative dynamics of the rapid progression of Parkinson's disease in patients with cardiovascular risk factors, impaired regulation of blood glucose levels, impaired uric acid metabolism, and inflammation (49). According to Huang Y.F. in patients with PD, the risk of stroke is higher than in the population (50). In the Siberian cohort of patients with Parkinson's disease, women with higher education, overweight, concomitant arterial hypertension, coronary heart disease, and dyslipidemia predominated. The predominance of females in the analyzed cohort of patients with Parkinson's disease is probably associated with regional features of a higher medical demand for females. The data obtained during the study are consistent with the results of other studies in various cohorts and in different parts of the world (51–53).

Smoking as a protective risk factor in Parkinson's disease is confirmed by a large number of scientific studies (54–57). Our data indirectly confirm and are consistent with previously published studies. The analysis found that 75% of respondents never smoke. The study found that household contact with various pesticides is relevant for 30% of respondents.

The Siberian cohort of patients with Parkinson's disease has its own peculiarities in the clinical picture of the disease, in questions of comorbidity, which necessitates further studies of various clinical, epidemiological, and genetic aspects of the disease using unified protocols to better understand the nature of the disease. The results presented in the article indicate the main directions of further deeper study of this pathology.

## Data Availability Statement

The raw data supporting the conclusions of this article will be made available by the authors, without undue reservation. Requests to access the datasets should be directed to Roman Andreevich Delov, delov_roman@mail.ru.

## Ethics Statement

The studies involving human participants were reviewed and approved by Ethics Committee of the Omsk State Medical University. The patients/participants provided their written informed consent to participate in this study.

## Author Contributions

AR: study conception and design. IG, EK, EM, and RD: acquisition of data. RD: analysis and interpretation of data, drafting of manuscript, and critical revision.

## Conflict of Interest

The authors declare that the research was conducted in the absence of any commercial or financial relationships that could be construed as a potential conflict of interest.

## References

[1] McCann H Stevens CH Cartwright H Halliday G M. α-Synucleinopathy phenotypes. Park Relat Disord. (2014) 20(Suppl.1): S62–7. 10.1016/S1353-8020(13)70017-8 24262191

[2] Levin OS Fedorova NV Parkinson's Disease. 6th Edn Moscow: MEDpress-inform (2016). p. 384.

[3] Guidelines for the diagnosis and treatment of Parkinson's disease /Illarioshkin SNOS Levin. 3rd Edn. (2019). p. 336.

[4] Gallagher DA Lees AJ Schrag A What are the most important nonmotor symptoms in patients with Parkinson's disease and are we missing them? Mov Disord. (2010) 25:2493–500. 10.1002/mds.23394 20922807

[5] Zis P Martinez-Martin P Sauerbier A Rizos A Sharma JC Worth PF. Non-motor symptoms burden in treated and untreated early Parkinson's disease patients: argument for non-motor subtypes. Eur J Neurol. (2015) 22:1145–50. 10.1111/ene.12733 25981492

[6] Chaudhuri KR Martinez-Martin P Brown RG Sethi K Stocchi F Odin P. The metric properties of a novel non-motor symptoms scale for Parkinson's disease: results from an international pilot study. Mov Disord. (2007) 22:1901–11. 10.1002/mds.21596 17674410

[7] Martinez-Martin P Rodriguez-Blazquez C Kurtis MM Chaudhuri KR NMSS Validation Group. The impact of non-motor symptoms on health-related quality of life of patients with Parkinson's disease. Mov Disord. (2011) 26:399–406. 10.1002/mds.23462 21264941

[8] Duncan GW Khoo TK Yarnall AJ O'Brien JT Coleman SY Brooks DJ. Health related quality of life in early Parkinson's disease: the impact of non-motor symptoms. Mov Disord. (2014) 29:195–202. 10.1002/mds.25664 24123307

[9] Bugalho P Lampreia T Miguel R Mendonça MD Caetano A Barbosa R. Non-motor symptoms in Portuguese Parkinson's disease patients: correlation and impact on quality of life and activities of daily Living. Sci Rep. (2016) 6:32267. 10.1038/srep32267 27573215 PMC5004191

[10] Goetz CG Fahn S Martinez-Martin P Poewe W Sampaio C Stebbins GT. Movement disorder society-sponsored revision of the unified parkinson's disease rating scale (MDS-UPDRS): process, format, and clinimetric testing plan. Mov Disord. (2007). 22:41–7. 10.1002/mds.21198 17115387

[11] Hoehn MM Yahr MD. Parkinsonism: onset, progression and mortality. Neurology. (1967) 17:427–42. 10.1212/WNL.17.5.427 6067254

[12] Hughes AJ Daniel SE Kilford L Lees AJ. Accuracy of clinical diagnosis of idiopathic Parkinson's disease: a clinico-pathological study of 100 cases. J Neurol Neurosurg Psychiatry. (1992) 55:181–4. 10.1136/jnnp.55.3.181 1564476 PMC1014720

[13] Nasreddine ZS Phillips NA Bedirian V Charbonneau S Whitehead V Collin I. The montreal cognitive assessment, MoCA: a brief screening tool for mild cognitive impairment. J Am Geriatr Soc. (2005) 53:695–9. 10.1111/j.1532-5415.2005.53221.x 15817019

[14] Moccia M Tedeschi E Ugga L Erro R Picillo M Caranci F. White matter changes and the development of motor phenotypes in *de novo* Parkinson's disease. J Neurol Sci. (2016) 367:215–9. 10.1016/j.jns.2016.06.015 27423590

[15] Ramani L Malek N Patterson J Nissen T Newman EJ. Relationship between [(123) I]-FP-CIT SPECT and clinical progression in Parkinson's disease. Acta Neurol Scand. (2017) 135:400–6. 10.1111/ane.12613 27255673

[16] Choi SM Kim BC Cho BH Kang KW Choi KH Kim JT. Comparison of two motor subtype classifications in *de novo* Parkinson's disease. Parkinsonism Relat Disord. (2018) 54:74–8. 10.1016/j.parkreldis.2018.04.021 29703644

[17] Alves G Larsen JP Emre M Wentzel-Larsen T Aarsland D. Changes in motor subtype and risk for incident dementia in Parkinson's disease. Mov Disord. (2006) 21:1123–30. 10.1002/mds.20897 16637023

[18] Muller B Larsen JP Wentzel-Larsen T Skeie GO Tysnes OB. Autonomic and sensory symptoms and signs in incident, untreated Parkinson's disease: frequent but mild. Mov Disord. (2011) 26:65–72. 10.1002/mds.23387 20925070

[19] Yuan YS Zhou XJ Tong Q Zhang L Zhang L Qi ZQ. Change in plasma levels of amino acid neurotransmitters and its correlation with clinical heterogeneity in early Parkinson's disease patients. CNS Neurosci Ther. (2013) 19:889–96. 10.1111/cns.12165 23981689 PMC6493594

[20] Poletti M Frosini D Pagni C Lucetti C Del Dotto P Tognoni G. The association between motor subtypes and alexithymia in *de novo* Parkinson's disease. J Neurol. (2011) 258:1042–5. 10.1007/s00415-010-5878-8 21188407

[21] Reinoso G Allen JC Jr Au WL Seah SH Tay KY Tan LC Clinical evolution of Parkinson's disease and prognostic factors affecting motor progression: 9-year follow-up study. Eur J Neurol. (2015) 22:457–63. 10.1111/ene.12476 24888502

[22] Konno T Deutschlander A Heckman MG Ossi M Vargas ER Strongosky AJ. Clinical evolution of Parkinson's disease and prognostic factors affecting motor progression: 9-year follow-up study. Eur J Neurol. (2018) 386:39–45. 10.1016/j.jns.2018.01.013 24888502

[23] Nicoletti A Mostile G Nicoletti G Arabia G Iliceto G Lamberti P. Comparison of clinical features among Parkinson's disease subtypes: a large retrospective study in a single center. J Neurol Sci. (2016) 263:888–94. 10.1007/s00415-016-8075-6 29406964

[24] Rajput AH Rajput ML Ferguson LW Rajput A. Baseline motor findings and Parkinson disease prognostic subtypes. Neurology. (2017) 89:138–43. 10.1212/WNL.0000000000004078 28592451 PMC5501934

[25] Auyeung M Tsoi TH Mok V Cheung CM Lee CN Li R. Ten year survival and outcomes in a prospective cohort of new onset Chinese Parkinson's disease patients. J Neurol Neurosurg Psychiatry. (2012) 83:607–11. 10.1136/jnnp-2011-301590 22362919

[26] Hiorth YH Lode K Larsen JP. Frequencies of falls and associated features at different stages of Parkinson's disease. Eur J Neurol. (2013) 20:160–6. 10.1111/j.1468-1331.2012.03821.x 22816560

[27] Ben-Joseph Aaron. Ethnic variation in the manifestation of parkinson's disease: a narrative review. J Parkinsons Dis. (2020) 10:31–45. 10.3233/JPD-191763 31868680 PMC7029316

[28] Li HJ Zhang MF Chen MX Hu AL Li JB Zhang B. Validation of the nonmotor symptoms questionnaire for Parkinson's disease: results from a Chinese pilot study. Int J Neurosci. (2015) 125:929–35. 10.3109/00207454.2014.986573 25387070

[29] Khedr EM El Fetoh NA Khalifa H Ahmed MA El Beh KM. Prevalence of non motor features in a cohort of Parkinson's disease patients. Clin Neurol Neurosurg. (2013) 115:673–7. 10.1016/j.clineuro.2012.07.032 22902078

[30] Bostantjopoulou S Katsarou Z Karakasis C Peitsidou E Milioni D Rossopoulos N. Evaluation of non-motor symptoms in Parkinson's disease: an underestimated necessity. Hippokratia. (2013) 17:214–9. 24470730 PMC3872456

[31] Martinez-Martin P Schapira AH Stocchi F Sethi K Odin P MacPhee G. Prevalence of nonmotor symptoms in Parkinson's disease in an international sett study using nonmotor symptoms questionnaire in 545 patients. Mov Disord. (2007) 22:1623–9. 10.1002/mds.21586 17546669

[32] Rodriguez-Violante M Cervantes-Arriaga A Villar-Velarde A Corona T. Relationship between the type and side of motor symptoms with the prevalence of non-motor symptoms in Parkinson's disease. Neurologia. (2011) 26:319–24. 10.1016/S2173-5808(11)70076-1 21315490

[33] Tanveer K Attique I Sadiq W Ahmad A. Non-motor symptoms in patients with Parkinson's disease: a cross-sectional survey. Cureus. (2018) 10:e3412. 10.7759/cureus.3412 30538900 PMC6281445

[34] Cosentino C Nuñez Y Torres L. Frequency of non-motor symptoms in Peruvian patients with Parkinson's disease. Arq Neuropsiquiatr. (2013) 71:216–9. 10.1590/0004-282X20130005 23588282

[35] Cheon SM Ha MS Park MJ Kim JW. Nonmotor symptoms of Parkinson's disease: Prevalence and awareness of patients and families. Parkinsonism Relat Disord. (2008) 14:286–90. 10.1016/j.parkreldis.2007.09.002 18042421

[36] Chaudhuri KR Prieto-Jurcynska C Naidu Y Mitra T Frades-Payo B Tluk S. The nondeclaration of nonmotor symptoms of Parkinson's disease to health care professionals: an international study using the nonmotor symptoms questionnaire. Mov Disord. (2010) 25:704–9. 10.1002/mds.22868 20437539

[37] Romenets SR Wolfson C Galatas C Pelletier A Altman R Wadup L. Validation of the non-motor symptoms questionnaire (NMS-Quest). Parkinsonism Relat Disord. (2012) 18:54–8. 10.1016/j.parkreldis.2011.08.013 21917501

[38] Wright Willis A Evanoff BA Lian M Criswell SR Racette BA. Geographic and ethnic variation in Parkinson disease: a population-based study of US Medicare beneficiaries. Neuroepidemiology. (2010) 34:143–51. 10.1159/000275491 20090375 PMC2865395

[39] GBD 2016 Parkinson's Disease Collaborators Global, regional, and national burden of Parkinson's disease, 1990–2016: a systematic analysis for the Global burden of disease study. Lancet Neurol. (2016) 17:939–53. 10.1016/S1474-4422(18)30295-3 PMC6191528 30287051

[40] Ongun N. Does nutritional status affect Parkinson's disease features and quality of life? PLoS ONE. (2018) 13:e0205100. 10.1371/journal.pone.0205100 30278074 PMC6168151

[41] Reeve A Simcox E Turnbull D. Ageing and Parkinson's disease: why is advancing age the biggest risk factor? Ageing Res Rev. (2014) 14:19–30. 10.1016/j.arr.2014.01.004 24503004 PMC3989046

[42] Alcalay RN Mirelman A Saunders-Pullman R Tang MX Mejia Santana H Raymond D. Parkinson disease phenotype in Ashkenazi Jews with and without LRRK2 G2019S mutations. Mov Disord. (2013) 28:1966–71. 10.1002/mds.25647 24243757 PMC3859844

[43] Huang T Shu Y Cai YD. Genetic differences among ethnic groups. BMC Genomics. (2015) 16:1093. 10.1186/s12864-015-2328-0 26690364 PMC4687076

[44] Branson CO Ferree A Hohler AD Saint-Hilaire MH Racial disparities in Parkinson disease: a systematic review of the literature. Adv Parkinsons Dis. (2016) 5:87–96. 10.4236/apd.2016.54011

[45] Rizzo G Copetti M Arcuti S Martino D Fontana A Logroscino G. Accuracy of clinical diagnosis of Parkinson disease: as systematic review and meta-analysis. Neurology. (2016) 86:566–76. 10.1212/WNL.0000000000002350 26764028

[46] Stanković I. Petrović I. Pekmezović T. Marković V. Stojković T. Dragašević-Mišković N.. Longitudinal assessment of autonomic dysfunction in early Parkinson's disease. Parkinsonism Relat Disord. (2019) 66:74–9. 10.1016/j.parkreldis.2019.07.008 31320275

[47] Valentino F Bartolotta TV Cosentino G Mastrilli S Arnao V Aridon P. Urological dysfunctions in patients with Parkinson's disease: clues from clinical and non-invasive urological assessment. BMC Neurol. (2018) 18:148. 10.1186/s12883-018-1151-z 30236066 PMC6146523

[48] Tibar H El Bayad K Bouhouche A Ait Ben Haddou EH Benomar A Yahyaoui M. Non-motor symptoms of Parkinson's disease and their impact on quality of life in a cohort of moroccan patients. Front Neurol. (2018) 9:170. 10.3389/fneur.2018.00170 29670566 PMC5893866

[49] Mollenhauer B Zimmermann J Sixel-Döring F Focke NK Wicke T Ebentheuer J. Baseline predictors for progression 4 years after Parkinson's disease diagnosis in the *De novo* Parkinson Cohort (DeNoPa). Move Disord. (2019) 34:67–77. 10.1002/mds.27492 30468694

[50] Huang YF Yeh CC Chou YC Hu CJ Cherng YG Shih CC. Stroke in Parkinson's disease. QJM. (2019) 112:269–74. 10.1093/qjmed/hcz015 30629254

[51] Malek N Lawton MA Swallow DM Grosset KA Marrinan SL Bajaj N. Vascular disease and vascular risk factors in relation to motor features and cognition in early Parkinson's disease. Move Disord. (2016) 31:1518–26. 10.1002/mds.26698 27324570 PMC5082556

[52] Fanciulli A Göbel G Ndayisaba JP Granata R Duerr S Strano S. Supine hypertension in Parkinson's disease and multiple system atrophy. Clin Auton Res. (2016) 26:97–105. 10.1007/s10286-015-0336-4 26801189

[53] Li HJ Yu Y Chen Y Liang HY. Vascular risk factors aggravate the progression of Parkinson's disease: a five-year follow-up study in Chinese patients. Int J Clin Exp Med. (2015) 8:9897–903. 26309021 PMC4537966

[54] Gallo V Vineis P Cancellieri M Chiodini P Barker RA Brayne C. Exploring causality of the association between smoking and Parkinson's disease. Int J Epidemiol. (2019) 48:912–25. 10.1093/ije/dyy230 30462234 PMC6659366

[55] Kim IY O'Reilly ÉJ Hughes KC Gao X Schwarzschild MA Hannan MT. Integration of risk factors for Parkinson disease in 2 large longitudinal cohorts. Neurolog. (2018) 90:e1646–53. 10.1212/WNL.0000000000005473 29643081 PMC5952970

[56] Breckenridge CB Berry C Chang ET Sielken RL Jr Mandel JS. Association between Parkinson's disease and cigarette smoking, rural living, well-water consumption, farming and pesticide use: systematic review and meta-analysis. PLoS ONE. (2016) 11:e0151841. 10.1371/journal.pone.0151841 27055126 PMC4824443

[57] Martino R Candundo H Lieshout PV Shin S Crispo J Barakat-Haddad C. Onset and progression factors in Parkinson's disease: a systematic review. Neurotoxicology. (2017) 61:132–41. 10.1016/j.neuro.2016.04.003 27058967

